# Elecsys CSF biomarker immunoassays demonstrate concordance with amyloid-PET imaging

**DOI:** 10.1186/s13195-020-00595-5

**Published:** 2020-03-31

**Authors:** James D. Doecke, Larry Ward, Samantha C. Burnham, Victor L. Villemagne, Qiao-Xin Li, Steven Collins, Christopher J. Fowler, Ekaterina Manuilova, Monika Widmann, Stephanie R. Rainey-Smith, Ralph N. Martins, Colin L. Masters, the AIBL Research Group

**Affiliations:** 1 Cooperative Research Council for Mental Health, Melbourne, Victoria 3052 Australia; 2 Australian E-Health Research Centre, CSIRO Health & Biosecurity, Level 5, 901/16 Royal Brisbane & Women’s Hospital, Brisbane, Queensland 4029 Australia; 3 Australian E-Health Research Centre, CSIRO, Parkville, Melbourne, Victoria 3052 Australia; 4 The Florey Institute of Neuroscience and Mental Health, The University of Melbourne, Parkville, Melbourne, Victoria 3010 Australia; 5 Department of Molecular Imaging and Therapy, Center for PET, Austin Health, Heidelberg, Victoria 3084 Australia; 6 Department of Medicine (RMH), The University of Melbourne, Parkville, Melbourne, Victoria 3052 Australia; 7 Roche Diagnostics GmbH, Nonnenwald 2, 82377 Penzberg, Germany; 8 Roche Diagnostics GmbH, Sandhoferstrasse 116, 68305 Mannheim, Germany; 9 Centre of Excellence for Alzheimer’s Disease Research and Care, School of Medical and Health Sciences, Edith Cowan University, Joondalup, Western Australia 6027 Australia; 10 Department of Biomedical Sciences, Macquarie University, North Ryde, New South Wales 2113 Australia; 11 School of Psychiatry and Clinical Neurosciences, University of Western Australia, Crawley, Western Australia 6009 Australia

**Keywords:** Alzheimer’s disease, Beta-amyloid, Cerebrospinal fluid, Concordance PET, Tau

## Abstract

**Background:**

β-amyloid (Aβ) positron emission tomography (PET) imaging is currently the only Food and Drug Administration-approved method to support clinical diagnosis of Alzheimer’s disease (AD). However, numerous research studies support the use of cerebrospinal fluid (CSF) biomarkers, as a cost-efficient, quick and equally valid method to define AD pathology.

**Methods:**

Using automated Elecsys® assays (Roche Diagnostics) for Aβ (1–42) (Aβ42), Aβ (1–40) (Aβ40), total tau (tTau) and phosphorylated tau (181P) (pTau), we examined CSF samples from 202 participants of the Australian Imaging, Biomarkers and Lifestyle (AIBL) study of ageing cohort, to demonstrate the concordance with pathological AD via PET imaging.

**Results:**

Ratios Aβ42/Aβ40, tTau/Aβ42 and pTau/Aβ42 had higher receiver operator characteristic—area under the curve (all 0.94), and greater concordance with Aβ-PET (overall percentage agreement ~ 90%), compared with individual biomarkers.

**Conclusion:**

Strong concordance between CSF biomarkers and Aβ-PET status was observed overall, including for cognitively normal participants, further strengthening the association between these markers of AD neuropathological burden for both developmental research studies and for use in clinical trials.

**Supplementary information:**

The online version of this article (10.1186/s13195-020-00595-5).

## Background

Alzheimer’s disease (AD) pathology is now recognised to evolve over an extended period before the onset of clinical symptoms [1], with homeostatic failure of the amyloid precursor protein cleavage appearing to be the primary pathogenic event [2–4]. The resulting accumulation of β-amyloid (Aβ) peptides into senile plaques is coupled with the degeneration of neurons, abnormal hyperphosphorylation of the tau protein and formation of tau neurofibrillary tangles [5, 6]. To date, visual assessment of Aβ positron emission tomography (PET) scans is the only Food and Drug Administration-approved method to support the clinical diagnosis of AD [7]. Whilst measurement of neocortical amyloid via PET is recognised as a core marker of disease pathological status, Aβ-PET imaging is costly and is not easily amenable for application to the wider community.

Inclusion of cerebrospinal fluid (CSF), total tau (tTau) and phosphorylated tau (181P) (pTau) in ratios with Aβ (1–42) (Aβ42) has been shown to improve biomarker performance, reflecting the strong relationship between the presence of both Aβ and tau pathologies in AD [8, 9]. Recent studies have shown good concordance between Aβ42 levels, and tTau/Aβ42 and pTau/Aβ42 ratios measured in CSF using a variety of platforms, including automated Elecsys® assays (Roche Diagnostics), and Aβ-PET outcome obtained using different radiotracers in diverse study cohorts [10, 11]. Furthermore, CSF biomarker status determined using predefined thresholds has been shown to predict clinical decline and progression to dementia in patients with mild cognitive impairment (MCI) [10, 12]. Accordingly, the use of CSF biomarkers to support AD diagnosis is recommended in recent research diagnostic guidelines published by the International Work Group 2 [13], as well as the National Institute on Ageing-Alzheimer’s Association (NIA-AA) biological framework for AD [14].

The present study aimed to provide additional evidence in support of the relationship between CSF biomarkers and the neuropathological Aβ-PET classification, thus further contributing to the NIA-AA research framework, which utilises the measurement of biomarkers to define an AD continuum [14, 15]. The relationship between CSF biomarkers Aβ42, Aβ (1–40) (Aβ40), tTau and pTau (and their ratios) with the neuropathological Aβ-PET classification status was evaluated both across the full clinical disease spectrum, and in cognitively normal controls from the highly characterised Australian Imaging, Biomarkers and Lifestyle (AIBL) study of ageing cohort.

## Methods

### Sample collection

This was a retrospective analysis of a sub-cohort of 202 participants from the AIBL study of ageing cohort [16], which utilised CSF samples and available neuropathological Aβ imaging data. Samples were included from participants who were diagnosed as either cognitively normal (CN; *n* = 140), with MCI (not necessarily due to AD, *n* = 33), with AD (*n* = 27) or with frontotemporal dementia (FTD; *n* = 2) via a cognitive and a subjective neuropsychological assessment. Prior written informed consent was obtained from all participants, and ethical approval was provided by all participating institutions.

### CSF collection

The CSF collection protocol has been published previously [17, 18] and is aligned with the Alzheimer’s Biomarkers Standardization Initiative [19]. Following an overnight fast, CSF was collected in the morning by lumbar puncture using a Temena (Polymedic®, EU) spinal needle micro-tip (22/27G × 103 mm; CAT 21922-27). Aseptic technique was adhered to at all times, with the participants sitting upright. CSF was collected by either gravity or aspiration into 15-mL polypropylene tubes (Greiner Bio-One188271). Samples were placed on ice immediately and kept between 2 °C and 8 °C during transport to the laboratory, and processed within 1 h. Samples were centrifuged at 2000×*g*, at 4 °C for 10 min and supernatant transferred to a fresh Greiner polypropylene tube and gently inverted. Aliquots were snap-frozen in 1-mL screw-cap 2D barcoded polypropylene Nunc Cryotubes (NUN374088) for long-term storage; samples were stored in liquid nitrogen vapour tanks until use and thawed once immediately before analysis.

### Immunoassays

The Elecsys β-Amyloid (1–42) CSF, Elecsys β-Amyloid (1–40) CSF, Elecsys Total Tau CSF and Elecsys Phospho-Tau (181P) CSF assays are electrochemiluminescence immunoassays, which can be run on cobas e 601, cobas e 602 and MODULAR ANALYTICS E170 analysers. The assays have measuring ranges of 200–1700 pg/mL (Aβ42), 0.011–39.540 ng/mL (Aβ40), 80–1300 pg/mL (tTau) and 8–120 pg/mL (pTau). The Elecsys β-Amyloid (1–40) assay is currently employed for research use only. Further information on each assay, including standardisation and analytical performance, can be found in previous publications [20–22]. Of the CSF biomarkers that were measured, 22% of the observations for Aβ42 were above the upper limit of the assay measuring range. Further information regarding how this was dealt with is shown in Additional file 1: Supplementary Methods.

### Amyloid-PET measurement

Aβ-PET imaging was performed with four different radiotracers: ^11^C-Pittsburgh compound B (PiB), ^18^F-NAV4694 (NAV), ^18^F-Flutemetamol (FLUTE) or ^18^F-Florbetapir (FBP). Methodology for each tracer has been previously described [23]. Briefly, standardised uptake values (SUVs) were calculated via summing spatially normalised PET images sampled using a narrow cortical regions of interest template (reducing possible noise from the measurement). The SUVs were then scaled to each tracer’s recommended reference regions to define the SUV ratio (SUVR). Reference region for NAV and PiB was the cerebellar cortex [24, 25], for FLUTE the pons [26] and for FBP the whole cerebellum [27]. Given that data from the NAV and PiB tracers have almost identical dynamic ranges, and only one participant had measurements from NAV alone, data from these tracers were combined and labelled as “NAV/PiB”. Quantitative SUVR values were dichotomised into Aβ-PET– or Aβ-PET+ based on each tracer-specific threshold (NAV/PiB: 1.4, FLUTE: 0.62 and FBP: 1.05). Briefly, for NAV/PiB, the binary Aβ-PET threshold was computed using a cluster analyses and compared with thresholds previously identified by Clark et al. [28], for FLUTE, Thurfjell et al. used a ROC method compared with post mortem results [29], and lastly for FBP, Clark et al. calculated the threshold to be the 95th percentile of the SUVR from young healthy controls (age 35–55 years and without cognitive impairment) [28].

### Population demographic comparisons

Population demographic characteristics (gender, age, apolipoprotein E [APOE] ε4 allele status, cognitive scores [the preclinical Alzheimer’s cognitive composite (PACC), Mini-Mental State Examination (MMSE) and Clinical Dementia Rating (CDR)], PET tracer frequency and clinical classification/diagnoses) were compared in Aβ-PET– and Aβ-PET+ groups using chi-squared test, independent-samples *t*-test and Mann–Whitney *U* test where appropriate.

### Comparisons of CSF biomarker means

Distribution of CSF biomarkers in groups with different PET status were compared using the Wilcoxon signed-rank test and generalised linear models accounting for covariates, including age, APOE ε4 allele status, gender and clinical classification/diagnosis. For the biomarker comparisons between Aβ-PET status, the two participants with FTD (and ultimately not on an AD pathway) were not included in statistical analyses.

### Biomarker threshold construction

Biomarker (both individual and ratio) thresholds were derived using the optimisation of Youden’s index [30] within receiver operating characteristic–area under the curve (ROC-AUC) analyses using dichotomised Aβ-PET status as an endpoint. In addition, for Aβ42/Aβ40, pTau/Aβ42 and tTau/Aβ42, which had clear bi-modal distributions, unsupervised thresholds were derived using two-component Gaussian mixture models (GMMs). Further information on the construction of the GMM’s and the derivation of their thresholds is shown in Additional file 1: Supplementary Methods.

### Concordance and performance of the CSF biomarkers with Aβ-PET

Elecsys CSF assay biomarkers and their respective ratios (Aβ42/Aβ40, tTau/Aβ42 and pTau/Aβ42) were analysed with respect to their concordance with Aβ-PET status, irrespective of clinical classification, and within sample with cognitively normal participants. The capability of individual CSF biomarkers and various ratios to distinguish participants classified as Aβ-PET+/− was assessed using ROC-AUC analyses. AUC values of individual biomarkers and biomarker ratios were compared using DeLong’s method [31]. Overall, positive and negative percentage agreements (OPA, PPA and NPA, respectively) with Aβ-PET status were calculated at all derived thresholds.

## Results

### Sample demographics and biomarker group-wise comparisons

**Fig. 1:**
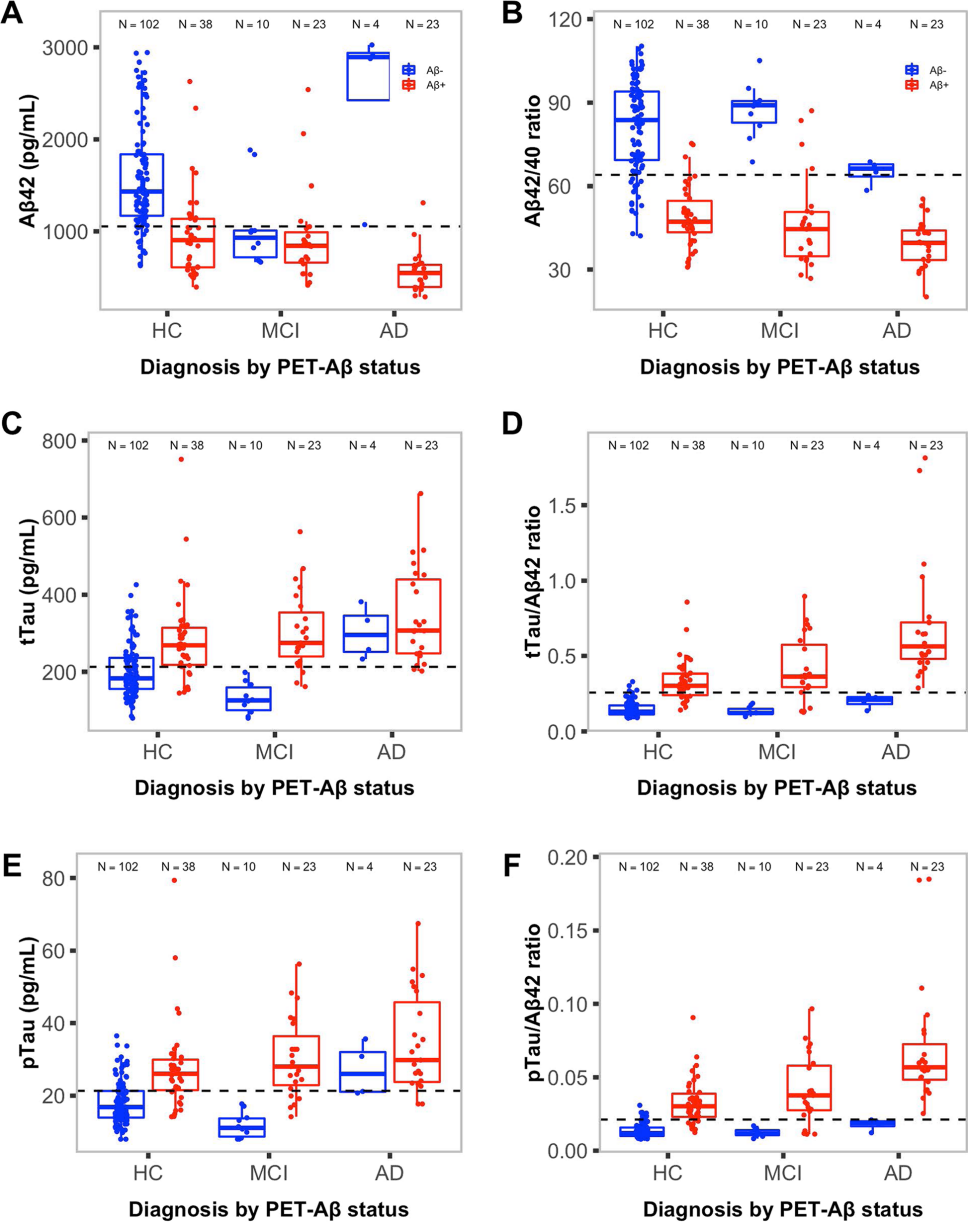
Box and whisker plots of CSF biomarkers by Aβ-PET status and clinical classification. **a** Aβ42, **b** Aβ42/Aβ40 ratio, **c** tTau, **d** tTau/Aβ42 ratio, **e** pTau and **f** pTau/Aβ42 ratio. Dashed lines represent threshold values for each CSF biomarker as calculated via ROC analyses. *Abbreviations*: *Aβ*, β-amyloid; *Aβ42*, β-amyloid (1–42); *Aβ42/Aβ40*, β-amyloid (1–42)/β-amyloid (1–40) ratio; *AD*, Alzheimer’s disease; *CSF*, cerebrospinal fluid; *HC*, healthy controls; *MCI*, mild cognitive impairment; *PET*, positron emission tomography; *pTau*, phosphorylated tau (181P); *pTau/Aβ42*, phosphorylated tau (181P)/β-amyloid (1–42) ratio; *ROC*, receiver operating characteristic; *tTau*, total tau; *tTau/Aβ42*, total tau/β-amyloid (1–42) ratio

Ninety participants were imaged using the NAV/PiB tracer, 70 with the FLUTE tracer and 42 with the FBP tracer; more participants were Aβ-PET– than Aβ-PET+ with each tracer. Overall, 38/140 (27%) CN participants, 23/33 (70%) participants with MCI and 23/27 (85%) participants with clinically diagnosed AD had Aβ-PET+ (Table 1). Both participants with FTD were Aβ-PET–. Participants who were Aβ-PET+ were more likely to be male (*P* = 0.03), older (*P* = 0.01) and be APOE ε4 allele status positive (*P* < 0.0001), with poorer cognitive scores (*P* < 0.0003). All CSF biomarker means and medians were significantly different between Aβ-PET groups (*P* < 0.0001; Additional file 2: Supplementary Table S1), with distributional differences between pathological and clinical subgroups shown in Fig. 1.

**Table 1:** Study population demographic characteristics, including comparisons between Aβ-PET groups

Characteristic	Total sample	Aβ-PET–	Aβ-PET+	*P* value
*n* (%)	202 (100)	118 (58)	84 (42)	–
Gender male, *n* (%)	100 (50)	51 (43)	49 (58)	0.0340
Mean (SD) age, years	73.5 (6.2)	72.5 (6.2)	74.8 (6.0)	0.0110
APOE ε4 allele status carriage, *n* (%)	64 (32)	24 (21)	40 (48)	< 0.0001
Mean (SD) PACC score	− 3.0 (6.8)	− 0.5 (4.2)	− 6.8 (8.1)	< 0.0001
Median (IQR) MMSE score	28 (4.0)	29 (2.0)	27 (4.2)	0.0002
Median (IQR) CDR score	0 (2.4)	0 (0)	0.5 (3.2)	0.0002
Tracer, *n* (%)				0.048
NAV/PiB	90 (44)	46 (23)	44 (22)	–
FLUTE	70 (35)	41 (20)	29 (14)	–
FBP	42 (21)	31 (15)	11 (6)	–
Clinical classification, *n* (%)				< 0.0001
CN	140 (70)	102 (51)	38 (19)	–
MCI	33 (16)	10 (5)	23 (11)	–
AD	27 (13)	4 (2)	23 (11)	–
FTD	2 (1)	2 (1)	0 (0)	–

*Abbreviations*: *Aβ*, β-amyloid; *AD*, Alzheimer’s disease; *APOE*, apolipoprotein E; *CDR*, Clinical Dementia Rating; *CN*, cognitively normal; *FBP*, ^18^F-florbetapir; *FLUTE*, ^18^F-flutemetamol; *FTD*, frontotemporal dementia; *IQR*, interquartile range; *MCI*, mild cognitive impairment; *MMSE*, Mini-Mental State Examination; *NAV*, ^18^F-NAV4694; *PACC*, Preclinical Alzheimer Cognitive Composite; *PET*, positron emission tomography; *PiB*, ^11^C-Pittsburgh compound B; *SD*, standard deviation

### CSF biomarker thresholds

Thresholds developed using the optimisation of Youden’s index based on the complete cohort for individual biomarkers Aβ42, tTau and pTau, and ratios Aβ42/Aβ40, tTau/Aβ42 and pTau/Aβ42, were derived as 1054 pg/mL, 213 pg/mL, 21.3 pg/mL, and 0.064, 0.258 and 0.0183, respectively. GMM analysis for the ratios resulted in the following thresholds: 0.0673 (95% confidence interval [CI] 0.0612–0.0798) for Aβ42/Aβ40, 0.165 (95% CI 0.150–0.187) for tTau/Aβ42 and 0.0159 (95% CI 0.0141–0.0184) for pTau/Aβ42. Biomarker distribution and goodness of fit are shown in Additional file 3: Supplementary Fig. S1 and Additional file 4: Supplementary Fig. S2.

### Concordance between CSF biomarkers and dichotomised Aβ-PET

**Fig. 2:**
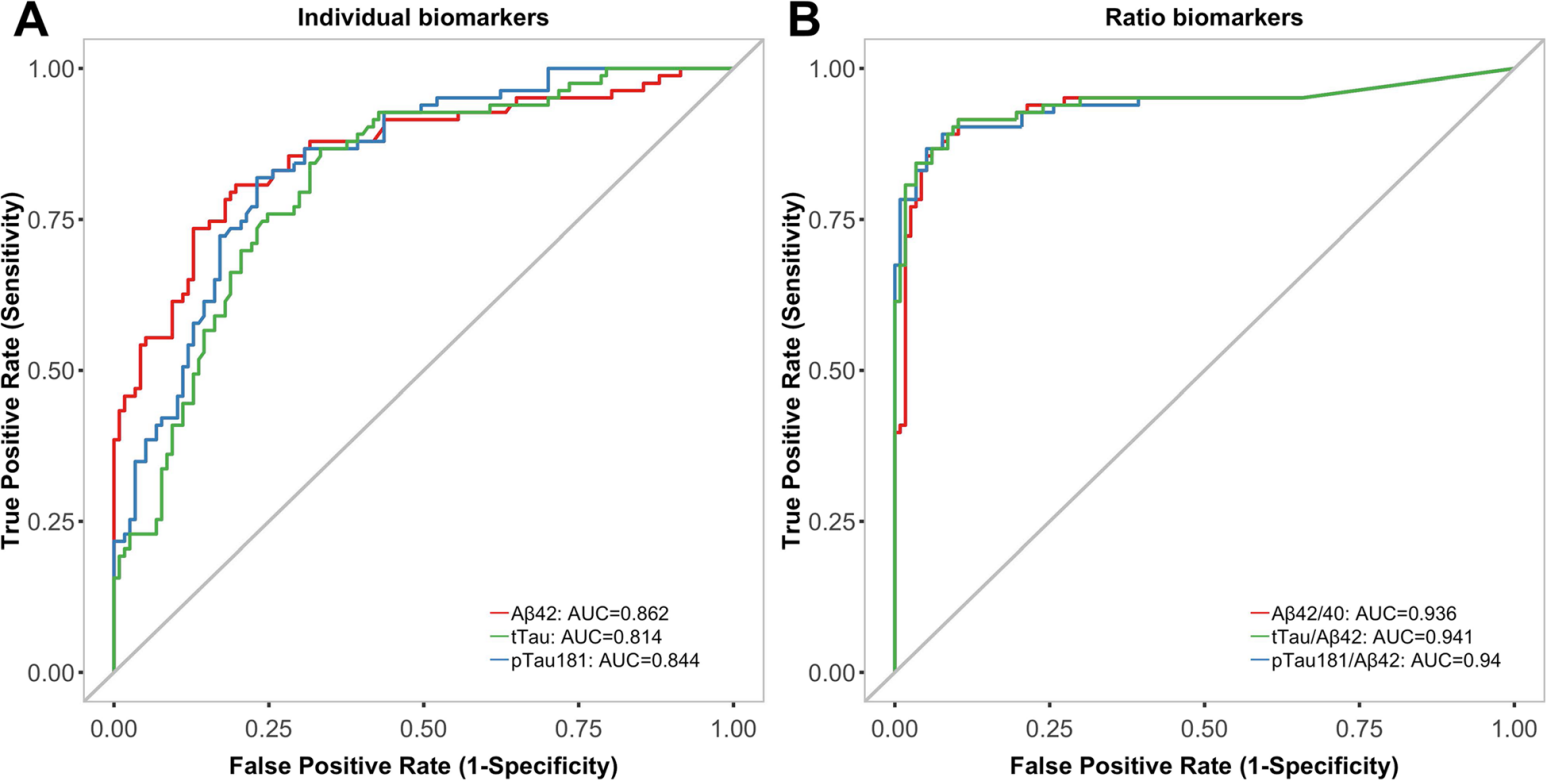
ROC curves of **a** individual CSF biomarkers and **b** biomarker ratios to predict Aβ-PET status. AUC statistics are presented for each biomarker. *Abbreviations*: *Aβ*, β-amyloid; *Aβ42*, β-amyloid (1–42); *Aβ42/Aβ40*, β-amyloid (1–42)/β-amyloid (1–40) ratio; *AUC*, area under the curve; *CSF*, cerebrospinal fluid; *PET*, positron emission tomography; *pTau*, phosphorylated tau (181P); *pTau/Aβ42*, phosphorylated tau (181P)/β-amyloid (1–42) ratio; *tTau*, total tau; *tTau/Aβ42*, total tau/β-amyloid (1–42) ratio

Aβ42 had the highest AUC among single biomarkers (0.86), followed by pTau (0.84) and tTau (0.81) (Fig. 2, Table 2). Compared with individual biomarkers, the ratios Aβ42/Aβ40, tTau/Aβ42 and pTau/Aβ42 demonstrated a considerably higher performance (*P* < 0.0001), which was similar for all ratios (AUC of 0.94).

**Table 2:** ROC curve results—CSF biomarkers for prediction of Aβ-PET status

Biomarker	AUC (95% CI)	Threshold	Optimisation method	PPA (%)	NPA (%)	OPA (%)
Aβ42	0.86 (0.81–0.92)	1054 pg/mL	Youden	81	81	81
tTau	0.81 (0.75–0.87)	213 pg/mL	Youden	86	66	75
pTau	0.84 (0.78–0.89)	21.3 pg/mL	Youden	81	77	79
Aβ42/Aβ40	0.94 (0.89–0.98)	0.064	Youden	90	90	90
		0.0673	GMM	92	88	90
tTau/Aβ42	0.94 (0.90–0.98)	0.258	Youden	83	97	91
		0.165	GMM	92	80	85
pTau/Aβ42	0.94 (0.90–0.98)	0.0183	Youden	90	91	91
		0.0159	GMM	90	83	86

*Abbreviations*: *Aβ*, β-amyloid; *Aβ42*, β-amyloid (1–42); *Aβ42/Aβ40*, β-amyloid (1–42)/β-amyloid (1–40) ratio; *AUC*, area under the curve; *CI*, confidence interval; *CSF*, cerebrospinal fluid; *GMM*, Gaussian mixture model; *NPA*, negative percentage agreement; *OPA*, overall percentage agreement; *PET*, positron emission tomography; *PPA*, positive percentage agreement; *pTau*, phosphorylated tau (181P); *pTau/Aβ42*, phosphorylated tau (181P)/β-amyloid (1–42) ratio; *ROC*, receiver operating characteristic; *tTau*, total tau; *tTau/Aβ42*, total tau/β-amyloid (1–42) ratio

Among the single biomarkers, Aβ42 had the highest concordance with Aβ-PET status at the threshold optimised using Youden’s index (OPA, PPA and NPA 81%, Table 2). Aβ42/Aβ40 and pTau/Aβ42 ratios outperformed single biomarkers and showed similar performance at the derived thresholds (OPA was 90%, with a PPA and an NPA close to 90%). Overall agreement to Aβ-PET status using unsupervised thresholds was similar for Aβ42/Aβ40 (90%), and slightly lower for pTau/Aβ42 (86%) and tTau/Aβ42 (85%) ratios than agreement when using thresholds derived by optimisation of Youden’s index (Table 2). The unsupervised threshold values were higher for Aβ42/Aβ40 and lower for the Tau/Aβ42 ratios than the optimised thresholds, resulting in slightly higher PPA and lower NPA.

### Concordance between CSF biomarkers and SUVR

**Fig. 3:**
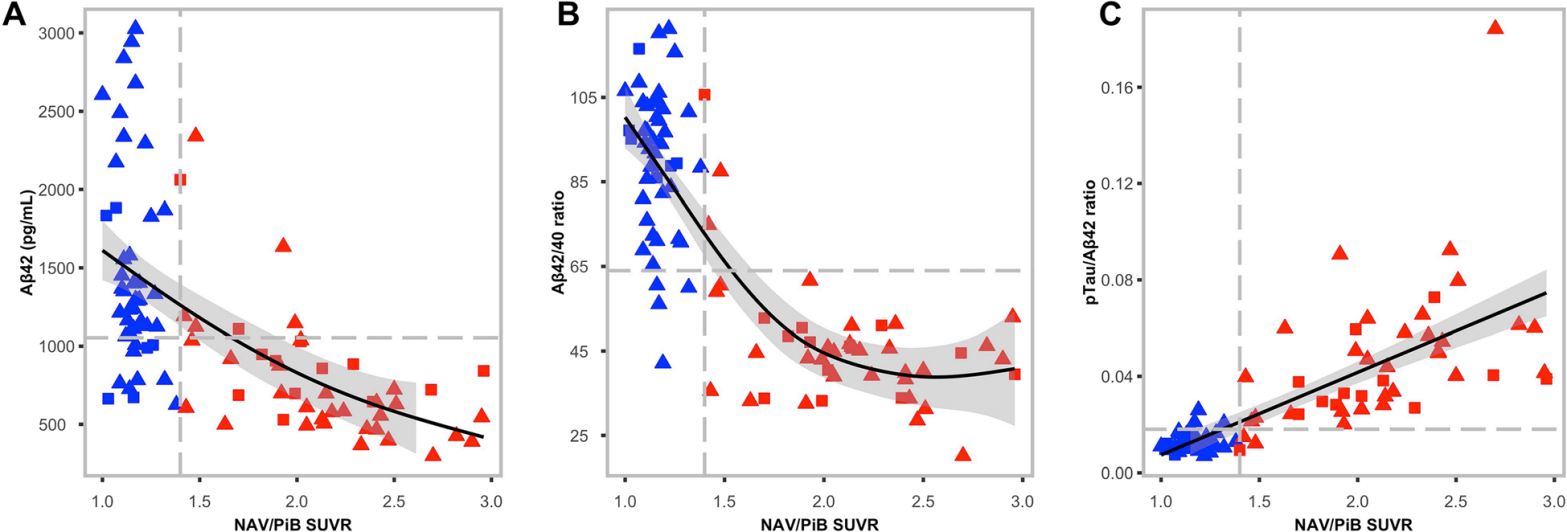
CSF biomarkers versus NAV/PiB SUVR for **a** Aβ42, threshold: 1054, **b** Aβ42/Aβ40 ratio, threshold: 64.0 (× 0.001) and **c** pTau/Aβ42 ratio,threshold: 0.018. Solid lines in plots **a** and **b** represent the non-linear relationship between CSF biomarkers and NAV/PiB SUVR. The solid line in plot **c** represents the linear relationship between NAV/PiB SUVR and pTau/Aβ42. Grey shaded areas represent the 95% CI around the solid line. Grey dashed lines represent thresholds for SUVR (vertical) and CSF (horizontal) biomarkers. Red symbols represent Aβ-PET+; blue symbols represent Aβ-PET–; circles represent CN participants; triangles represent participants with MCI; squares represent participants with AD. *Abbreviations*: *Aβ*, β-amyloid; *Aβ42*, β-amyloid (1–42); *Aβ42/Aβ40*, β-amyloid (1–42)/β-amyloid (1–40) ratio; *AD*, Alzheimer’s disease; *CI*, confidence interval; *CN*, cognitively normal; *CSF*, cerebrospinal fluid; *MCI*, mild cognitive impairment; *NAV*, ^18^F-NAV4694; *PET*, positron emission tomography; *PiB*, ^11^C-Pittsburgh compound B; *pTau/Aβ42*, phosphorylated tau (181P)/β-amyloid (1–42) ratio; *SUVR*, standardised uptake value ratio

We investigated the relationship between the insoluble aggregated form of Aβ (via quantitative NAV/PiB SUVR) and the soluble form of Aβ via CSF biomarkers Aβ42, the Aβ42/Aβ40 ratio and the pTau/Aβ42 ratio. Given the nature of the two different pools of Aβ, we performed non-linear regression to estimate the relationship. Using threshold lines for the CSF biomarker and SUVR, Fig. 3 shows high concordance between CSF and Aβ-PET status, and the specific relationships between SUVR and CSF biomarkers. Additional file 5: Supplementary Fig. S3 demonstrates qualitatively similar relationships for both FLUTE and FBP tracers.

### Correlation structure between CSF Aβ42 and CSF tau

**Fig. 4:**
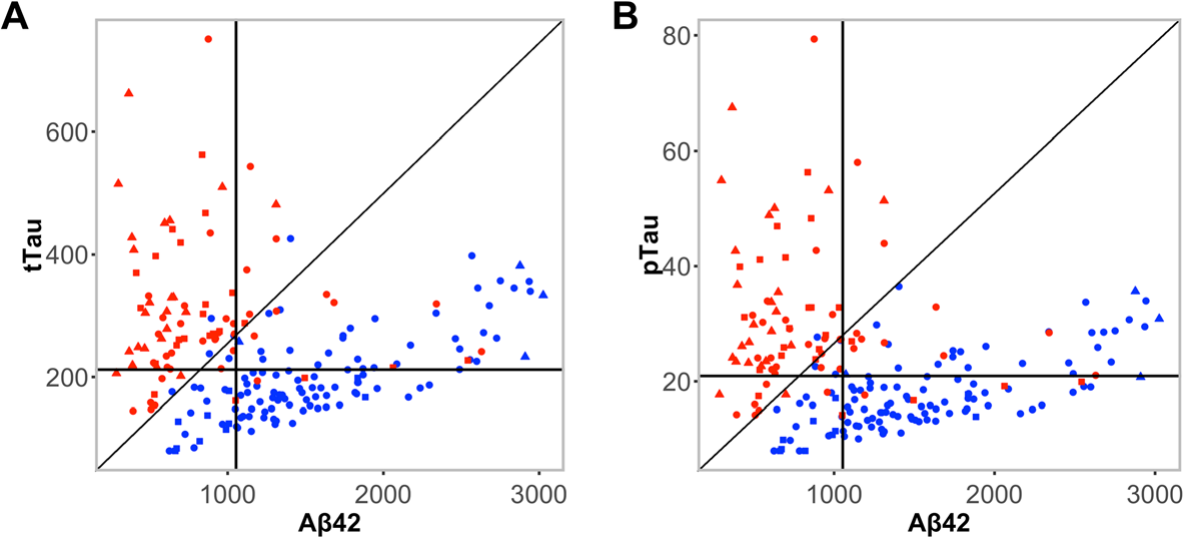
CSF biomarker relationships for **a** tTau versus Aβ42 and **b** pTau versus Aβ42. Diagonal lines represent the split between the two clusters. Horizontal and vertical lines represent the cut-offs. Red symbols represent Aβ-PET+; blue symbols represent Aβ-PET–; circles represent cognitively normal participants; triangles represent participants with MCI; squares represent participants with AD. *Abbreviations*: *Aβ*, β-amyloid; *Aβ42*, β-amyloid (1–42); *AD*, Alzheimer’s disease; *CSF*, cerebrospinal fluid; *MCI*, mild cognitive impairment; *PET*, positron emission tomography; *pTau*, phosphorylated tau (181P); *tTau*, total tau

Scatter plots for Aβ42 versus tTau and Aβ42 versus pTau showed two clusters (Fig. 4). The majority of Aβ-PET– participants had values aligning close to the *x*-axis, whilst those participants who were Aβ-PET+ had values aligning close to the *y*-axis (Fig. 4a, b). Diagonal lines corresponding to the thresholds derived by the optimisation of Youden’s index for ratios tTau/Aβ42 (Fig. 4a) and pTau/Aβ42 (Fig. 4b), clearly separating participants with positive and negative Aβ-PET status. Apparent clusters (red for Aβ-PET+ and blue for Aβ-PET–) demonstrated the ability of the marker to align with neuropathological amyloid load.

### PET concordance analysis split by tracer

Given that four different tracers were used within this study and that the relationship between PET tracer and correlated biomarker has previously been shown to vary by tracer [32, 33], all binary PET comparisons were performed using tracer-specific PET status. Whilst the distribution of Aβ-PET+/− participants was different between tracers, results of ROC-AUC analysis were similar (Additional file 6: Supplementary Table S2), with small differences in performance possibly due to the limited sample sizes.

### PET concordance analysis in CN samples

Biomarker thresholds developed based on the optimisation of Youden’s index were 1046 pg/mL for Aβ42, and 0.064, 0.184 and 0.0186 for Aβ42/Aβ40, tTau/Aβ42 and pTau/Aβ42, respectively. Agreement OPA was 83% for Aβ42 and was 89%, 89% and 90% for Aβ42/Aβ40, tTau/Aβ42 and pTau/Aβ42, respectively (Additional file 7: Supplementary Table S3). We observed a decrease in PPA and NPA of only 1% for Aβ42/Aβ40 and pTau/Aβ42 ratios, with very similar optimised threshold values compared with the whole population. The threshold for tTau/Aβ42 was slightly lower for CN participants, resulting in a higher PPA (92% vs 83% in the whole population) and lower NPA (87% vs 97% in the whole population).

## Discussion

The current study shows high concordance between neuropathological AD CSF biomarkers and Aβ-PET classification in both the overall sub-cohort of AIBL, and in the subgroup of CN participants.

When comparing Aβ-PET concordance for the CSF biomarker ratios across the complete cohort, irrespective of clinical classification, the GMM unsupervised thresholds resulted in higher PPA and lower NPA than the ROC-AUC thresholds. The unsupervised thresholds distinguish AD-like from non-AD-like biomarker profiles. In our study, 90% of participants with AD-like CSF in the Aβ42/Aβ40 ratio were Aβ-PET+, and 90% of participants with non-AD-like CSF in the Aβ42/Aβ40 ratio were Aβ-PET–. The corresponding numbers for the pTau/Aβ42 ratio were 90% and 91%, and for the tTau/Aβ42 ratio were 83% and 97%. Only 3–10% of patients with non-AD-like biomarker profiles were Aβ-PET+, but 10–17% of patients with pathological CSF biomarker profiles were Aβ-PET–. This finding is consistent with the notion that CSF biomarkers are able to identify participants at risk of developing clinical AD much earlier than Aβ-PET. However, in our cohort, this notion could not be confirmed due to the very small number of participants whose diagnosis changed during the follow-up period.

CSF Tau/Aβ42 and Aβ42/Aβ40 ratios demonstrated greater concordance with Aβ-PET status compared with individual biomarkers. In particular, OPA of the Tau/Aβ42 (90%) and Aβ42/Aβ40 (90%) ratios outperformed Aβ42 alone (81%). These results are consistent with previously published PET concordance studies using Elecsys assays. For example, concordance analysis with visual PET outcome in a subset of patients with mild cognitive symptoms from the Swedish BioFINDER cohort showed that the CSF tTau/Aβ42 and pTau/Aβ42 ratios have a higher OPA (90%) compared with Aβ42 alone (80%) [10]. Similarly, in a Korean cohort of patients with AD, concordance with NAV/PiB, PET SUVR was improved using the tTau/Aβ42 ratio (OPA, 92.5) over Aβ42 alone (OPA, 85.2%) [34]. Consistent with these findings, a recent roadmap for AD biomarkers also identified the greater diagnostic utility of CSF biomarker ratios [15]. Possible explanations for the better performance of CSF biomarker ratios over Aβ42 alone have been discussed previously [10]. Briefly, Tau/Aβ42 ratios combine the two core biomarkers of the principal pathological processes, underlying AD into a single marker; combining measurements of two different proteins may compensate for natural fluctuations in the levels of each protein; and the temporal profile of Aβ42 and tau biomarkers differ, with Aβ42 considered to be an earlier metric of disease development than tau [35].

Similar to results from Schindler et al. [11], in cognitively normal patients assessed using PET with a cut-off of 1.42, performance of the pTau and tTau ratios with Aβ42 was very close to that of the Aβ42/40 ratio (OPA AIBL Aβ42/40: 90%, OPA ADRC Aβ42/40: 86%; OPA AIBL pTau/Aβ42: 91%, OPA ADRC pTau/Aβ42: 89%; OPA AIBL tTau/Aβ42: 91%, OPA ADRC tTau/Aβ42: 87%). Given the stage at which a participant is measured as Aβ-PET+, it is likely that the amyloid accumulation has caused synaptic damage, causing Tau to be released and accumulate into tangles (as a secondary event). As such the CSF Tau biomarkers are increasing and the CSF Aβ42 is simultaneously decreasing.

Given the inherent relationship between CSF biomarkers and Aβ-PET status, we investigated the relationship between NAV/PiB SUVR and the CSF biomarkers Aβ42, Aβ42/Aβ40 ratio, tTau/Aβ42 ratio and pTau/Aβ42 ratio. The Tau/Aβ42 ratios demonstrated a slightly higher overall agreement than the Aβ42/Aβ40 ratio, albeit not significant, and both ratios outperformed Aβ42 alone. Similar relationships were observed using FLUTE and FBP tracers.

Good concordance between CSF biomarkers and Aβ-PET classification was also observed in the subset of participants with normal cognition, and the degree of concordance was equivalent to that observed in the whole study population, differing only by approximately 1% in terms of NPA and PPA for the majority of biomarkers. These results support the opinion that AD pathogenesis is progressive and continuous, and changes in biomarkers occur prior to the onset of clinical symptoms. Our findings in CSF samples from AIBL study participants are consistent with those previously reported in other clinical cohorts, including BioFINDER and Alzheimer’s Disease Neuroimaging Initiative (ADNI). Given the large proportion of cognitively normal elderly in the AIBL population, as compared with BioFINDER and ADNI, which had larger sample sizes in their MCI and AD populations, it is interesting that similar results overall were identified across these groups. This adds to the current knowledge that these neuropathological CSF biomarkers are highly predictive of amyloid plaques, irrespective of clinical stage.

This study presents research-based thresholds for CSF biomarkers for the separation of Aβ-PET groups similar to that of Hansson et al. [10] and Schindler et al. [11]; however, due to differences in sample handling and pre-analytical procedures, which may potentially affect measured CSF biomarker levels and biomarker thresholds [36, 37], the thresholds are not directly comparable. Whilst this presents a problem for direct comparison of thresholds, the performance of this and other studies all point towards strong agreement between the soluble Aβ and Tau as measured by CSF along with the measurement of the insoluble amyloid as measured by PET MRI.

Limitations of the present study include that PET SUVR is a proxy for histopathology, which is the current “gold standard” for establishing amyloid status. Additionally, the current research study used several radiotracers; this is however, reflective of both true clinical practice and research studies whereby funding constraints affect the ability to scan patients/participants. Reassuringly, results of the ROC-AUC analyses here were similar across the tracers. Retrospective samples were used from a small subset of participants, potentially reducing the reliability of our findings. The NPA and PPA values calculated at cut-offs derived by optimisation of Youden’s index may be overoptimistic and should be validated in an independent data set.

A small number of participants who were diagnosed with AD-dementia or MCI did not have both tau and amyloid pathology (e.g. negative Aβ-PET, normal CSF Aβ42 and abnormal CSF tau levels), and thus neuropathological AD was likely not the cause of their cognitive impairment. Of interest, two participants with FTD were both Aβ-PET−, demonstrating the absence of AD pathology in this type of dementia. As a strength, the study was therefore representative of a true population and shows the important role of biomarkers in differentiating AD from other forms of neurodegenerative diseases. Finally, the study is based on a single longitudinal research cohort, employing uniform approaches to all aspects, including CSF specimen handling, leaving some uncertainty about the generalisability of the findings to more diverse populations with a higher likelihood of less systematic technical rigour in relation to the biomarkers.

## Conclusion

The AD CSF biomarkers showed high concordance with Aβ-PET status in a cohort of individuals from the AIBL study. All three biomarker ratios (Aβ42/Aβ40, tTau/Aβ42 and pTau/Aβ42) demonstrated superior performance to Aβ42 alone. These results further strengthen evidence supporting the potential diagnostic utility of CSF biomarkers, including the Elecsys platform biomarkers for identification of individuals at risk of AD in prodromal/preclinical populations with normal cognition and early symptomatic patients, as well as for participant selection in therapeutic trials.

## Supplementary information

Additional file 1: Supplementary Methods. Information on handling of Aβ42 values above the measurement range and additional statistical information.

Additional file 2: Table S1. CSF biomarker univariate assessment with Aβ status. Two participants with FTD were not included in statistical analyses.

Additional file 3: Figure S1. Threshold determination using mixture modelling for the biomarkers (A, B) Aβ42/Aβ40, (C, D) pTau/Aβ42 and (E, F) tTau/Aβ42.

Additional file 4: Figure S2. Threshold determination using mixture modelling for the biomarkers (A, B) Aβ42, (C, D) pTau and (E, F) tTau.

Additional file 5: Figure S3. CSF biomarkers versus PET SUVR for: (A) Aβ42 versus FLUTE SUVR, (B) Aβ42/Aβ40 versus FLUTE SUVR, (C) pTau/Aβ42 versus FLUTE SUVR, (D) Aβ42 versus FBP SUVR, (E) Aβ42/Aβ40 versus FBP SUVR and (F) pTau/Aβ42 versus FBP SUVR.

Additional file 6: Table S2. Results of ROC-AUC analysis – CSF biomarkers to predict Aβ-PET status, by PET tracer.

Additional file 7: Table S3. Results of ROC-AUC analysis – CSF biomarkers to predict Aβ-PET status in CN individuals.

## Data Availability

Anonymised data is available upon request from any qualified investigator for the sole purpose of replicating procedures and results presented in the article.

## Abbreviations

Aβ: β-amyloid
AD: Alzheimer’s disease
ADNI: Alzheimer’s Disease Neuroimaging Initiative
AIBL: Australian Imaging, Biomarkers and Lifestyle
APOE: Apolipoprotein E
AUC: Area under the curve
CDR: Clinical Dementia Rating
CI: Confidence interval
CN: Cognitively normal
CSF: Cerebrospinal fluid
FBP: ^18^F-Florbetapir
FLUTE: ^18^F-Flutemetamol
FTD: Frontotemporal dementia
GMM: Gaussian mixture model
HC: Healthy controls
IQR: Interquartile range
MCI: Mild cognitive impairment
MMSE: Mini-Mental State Examination
NAV: ^18^F-NAV4694
NIA-AA: National Institute on Ageing-Alzheimer’s Association
NPA: Negative percentage agreement
OPA: Overall percentage agreement
PACC: Preclinical Alzheimer’s cognitive composite
PET: Positron emission tomography
PiB: ^11^C-Pittsburgh compound B
PPA: Positive percentage agreement
pTau: Phosphorylated tau (181P)
QQ: Quantile-quantile
ROC: Receiver operating characteristic
SD: Standard deviation
SUV: Standardised uptake value
SUVR: SUV ratio
tTau: Total tau
Aβ40: β-amyloid (1–40)
Aβ42: β-amyloid (1–42)
