# Supplementary material for: Elecsys CSF biomarker immunoassays demonstrate concordance with amyloid-PET imaging
Source: Alzheimers Res Ther. 2020 Mar 31;12:36. doi: 10.1186/s13195-020-00595-5 (PMC7110644; doi:10.1186/s13195-020-00595-5)
Supplement: Supplementary file 1 — Supplementary Methods. Information on handling of Aβ42 values above the measurement range and additional statistical information. [file 13195_2020_595_MOESM1_ESM.pdf]

Additional file 1: Supplementary Methods

Handling of Aβ42 values above the measurement range

Aβ42 measurements above the upper limit of measuring range (1700 pg/mL)

For the Aβ42 assay, 22% of observations were above the upper limit of the assay measuring range. For data visualisation and calculation of unsupervised thresholds using GMMs, Aβ42 concentrations above the measuring range were extrapolated using the raw signals and calibration curve. For the ROC-AUC analysis, Aβ42 values above the measuring range were set to the technical limits. For ratios Aβ42/Aβ40, tTau/Aβ42 and pTau/Aβ42, the following rule was used: if Aβ42 was above the measuring range, the participant was considered as negative independently from the tau (or Aβ40) value. Otherwise, the ratio value was calculated and compared with the threshold. To incorporate this rule in the ROC-AUC analysis, all ratio values were set to the minimum (maximum for Aβ42/Aβ40) ratio value if the corresponding Aβ42 value was above the measuring range. Values for Aβ40 were used only to define the ratio Aβ42/Aβ40, and not for any further analyses.

Threshold construction using Gaussian mixture models (GMMs)

The term "unsupervised" emphasises that the GMM thresholds are calculated based on biomarker distribution without using any information about PET status. For each biomarker ratio, an unsupervised threshold was established as a crossing point of equally weighted Gaussian density functions fitted to the distributions in AD-like and non-AD-like sub-populations. Details on the use of GMM to separate AD-like and non-AD-like participants can be found in De Meyer et al. [1] and Buchhave et al. [2]. The models were fitted using the R package mixtools 1.1.0 [3].

The goodness of fit of the parametric model used for threshold determination was assessed by a quantile-quantile (Q-Q) diagram. Random numbers were generated using the fitted model, and the resulting theoretical quantiles were plotted against the observed quantiles of the biomarker concentrations. Significance of quantile differences were assessed by calculation of a simultaneous tolerance bound with 95% coverage [4]. To improve fit, log-transformation was applied to pTau/Aβ42 and tTau/Aβ42 distributions.

References

1. De Meyer G, Shapiro F, Vanderstichele H, Vanmechelen E, Engelborghs S, De Deyn PP, et al. Alzheimer's Disease Neuroimaging Initiative. Diagnosis independent Alzheimer disease biomarker signature in cognitively normal elderly people. Arch Neurol. 2010;67:949–56.

2. Buchhave P, Minthon L, Zetterberg H, Wallin AK, Blennow K, Hansson O. Cerebrospinal fluid levels of β-amyloid 1-42, but not of tau, are fully changed already 5 to 10 years before the onset of Alzheimer dementia. Arch Gen Psychiatry. 2012;69:98–106.

3. Benaglia T, Chauveau D, Hunter DR, Young DS. Mixtools: an R package for analyzing finite mixture models. J Stat Softw. 2009;32:1–29.

4. Schützenmeister A, Jensen U, Piepho H-P. Checking normality and homoscedasticity in the general linear model using diagnostic plots. Commun Stat Simul Comput. 2012;41:141–54.
