# Supplementary material for: Elecsys CSF biomarker immunoassays demonstrate concordance with amyloid-PET imaging
Source: Alzheimers Res Ther. 2020 Mar 31;12:36. doi: 10.1186/s13195-020-00595-5 (PMC7110644; doi:10.1186/s13195-020-00595-5)
Supplement: Supplementary file 2 — CSF biomarker univariate assessment with Aβ status. Two participants with FTD were not included in statistical analyses. [file 13195_2020_595_MOESM2_ESM.pdf]

Additional file 2: Supplementary Table S1 CSF biomarker univariate assessment with Aβ status

Biomarker	Aβ-PET–, *n*	Aβ-PET+, *n*	Mean (SD) Aβ-PET–	Mean (SD) Aβ-PET+	*P* value[a]	Adjusted *P* value[b]
Aβ40	118	84	17.43 (5.60)	17.60 (5.42)	0.968	0.704
Aβ42	118	84	1559 (607)	843 (463)	< 0.0001	< 0.0001
pTau	118	84	18.0 (6.35)	30.3 (12.5)	< 0.0001	< 0.0001
tTau	118	84	201 (72.7)	306 (114)	< 0.0001	< 0.0001
Aβ42/Aβ40	118	84	89.5 (17.3)	47.2 (16.4)	< 0.0001	< 0.0001
tTau/Aβ42	118	84	0.137 (0.0491)	0.452 (0.293)	< 0.0001	< 0.0001
pTau/Aβ42	118	84	0.0124 (0.00450)	0.0451 (0.0307)	< 0.0001	< 0.0001

[a]Univariate unadjusted *P* values from Wilcoxon comparison of ranks

[b]Adjusted *P* values for the generalised linear models accounting for covariates, including age, APOE ε4 allele status, gender and clinical classification; 2 FTD patients were not included in the calculation of these adjusted *P* values

Please note: Two participants with FTD were not included in any further statistical analyses

Abbreviations: *Aβ*, β-amyloid; *Aβ40*, β-amyloid (1–40); *Aβ42*, β-amyloid (1–42); *Aβ42/Aβ40*, β-amyloid (1–42)/β-amyloid (1–40) ratio; *APOE*, apolipoprotein E; *CSF*, cerebrospinal fluid; *FTD*, frontotemporal dementia; *PET*, positron emission tomography;

pTau, phosphorylated tau (181P); *pTau/Aβ42*, phosphorylated tau (181P)/β-amyloid (1–42) ratio; *SD*, standard deviation;

t-Tau, total tau; *tTau/Aβ42*, total tau/β-amyloid (1–42) ratio
