# Supplementary material for: Elecsys CSF biomarker immunoassays demonstrate concordance with amyloid-PET imaging
Source: Alzheimers Res Ther. 2020 Mar 31;12:36. doi: 10.1186/s13195-020-00595-5 (PMC7110644; doi:10.1186/s13195-020-00595-5)
Supplement: Supplementary file 3 — Threshold determination using mixture modelling for the biomarkers (A, B) Aβ42/Aβ40, (C, D) pTau/Aβ42 and (E, F) tTau/Aβ42. [file 13195_2020_595_MOESM3_ESM.pdf]

Additional file 3: Supplementary Fig. S1 Threshold determination using mixture modelling for the biomarkers (A, B) Aβ42/Aβ40, (C, D) pTau/Aβ42 and (E, F) tTau/Aβ42. Left panel (parts A, C and E): biomarker distributions and the results of GMM fit. Red and blue solid curves represent densities of Gaussian distributions fitted to the AD-like (red) and non-AD-like (blue) populations. Vertical grey dashed lines correspond to the unsupervised thresholds. Right panel (parts B, D and F): goodness of the model fit: the QQ diagrams compare theoretical and observed quantiles. Blue dashed lines: simultaneous tolerance bounds with 95% coverage [1]. *Abbreviations*: *Aβ42/Aβ40*, β-amyloid (1–42)/β-amyloid (1–40) ratio; *AD*, Alzheimer's disease; *GMM*, Gaussian mixture model; *QQ*, quantile-quantile; *pTau/Aβ42*, phosphorylated tau (181P)/β-amyloid (1–42) ratio; *tTau/Aβ42*, total tau/β-amyloid (1–42) ratio

Reference

[1] Schützenmeister A, Jensen U, Piepho H-P. Checking normality and homoscedasticity in the general linear model using diagnostic plots. Commun Stat Simul Comput. 2012;41:141-54

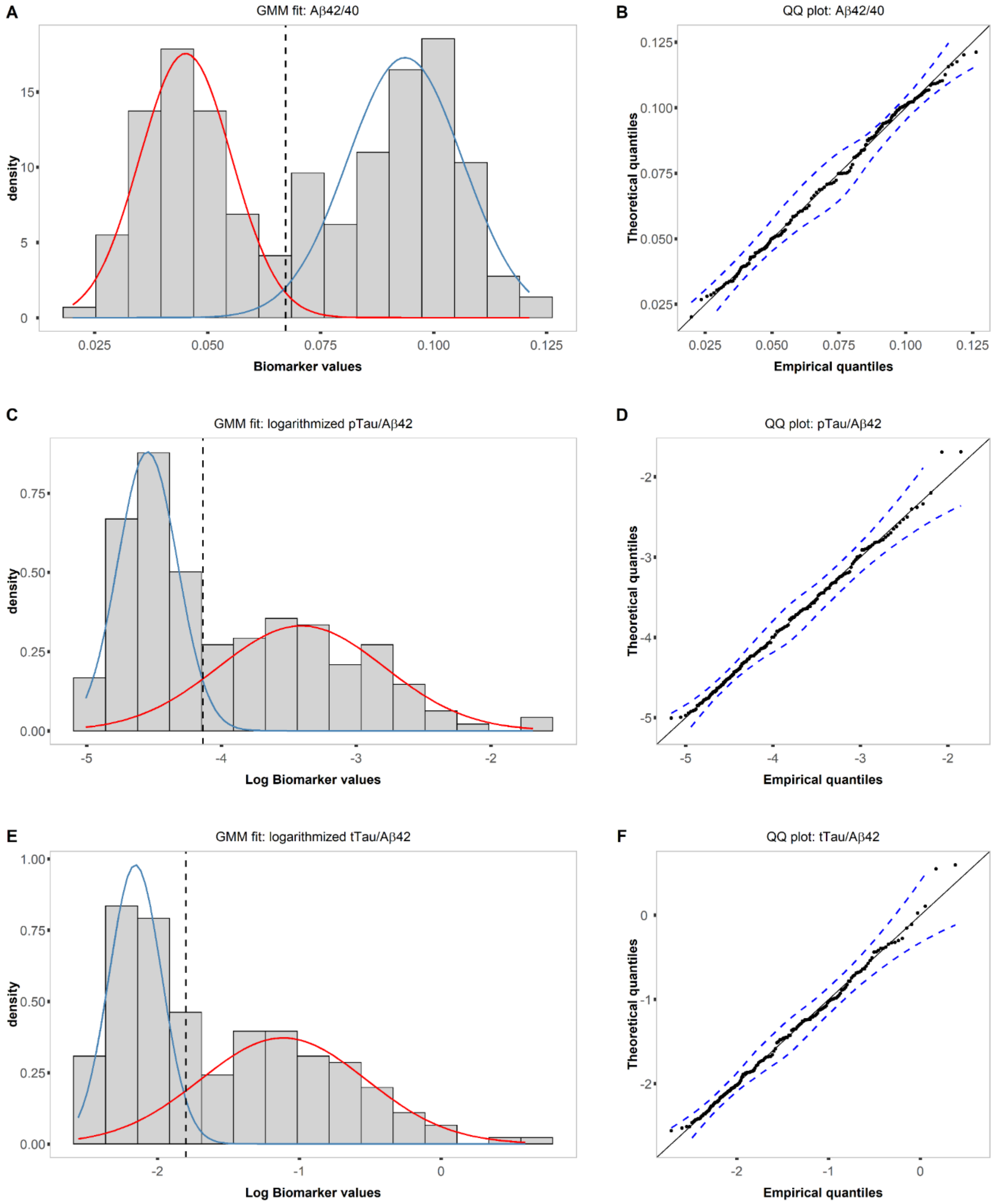

A
GMM fit: Aβ42/40
density
0
5
10
15
0.025
0.050
0.075
0.100
0.125
Biomarker values
B
QQ plot: Aβ42/40
Theoretical quantiles
0.025
0.050
0.075
0.100
0.125
Empirical quantiles
C
GMM fit: logarithmized pTau/Aβ42
density
0.00
0.25
0.50
0.75
-5
-4
-3
-2
Log Biomarker values
D
QQ plot: pTau/Aβ42
Theoretical quantiles
-5
-4
-3
-2
Empirical quantiles
E
GMM fit: logarithmized tTau/Aβ42
density
0.00
0.25
0.50
0.75
1.00
-2
-1
0
Log Biomarker values
F
QQ plot: tTau/Aβ42
Theoretical quantiles
-2
-1
0
Empirical quantiles
