# Supplementary material for: Elecsys CSF biomarker immunoassays demonstrate concordance with amyloid-PET imaging
Source: Alzheimers Res Ther. 2020 Mar 31;12:36. doi: 10.1186/s13195-020-00595-5 (PMC7110644; doi:10.1186/s13195-020-00595-5)
Supplement: Supplementary file 4 — Threshold determination using mixture modelling for the biomarkers (A, B) Aβ42, (C, D) pTau and (E, F) tTau. [file 13195_2020_595_MOESM4_ESM.pdf]

Additional file 4: Supplementary Fig. S2

Threshold determination using mixture modelling for the biomarkers (A, B) Aβ42, (C, D) pTau and (E, F) tTau. Left panel (parts A, C and E): biomarker distributions and the results of Log-normal fit. Black solid curves represent the log-normal distribution fit. Right panel (parts B, D and F): goodness of the model fit: the QQ diagrams compare theoretical and observed quantiles. Blue dashed lines: simultaneous tolerance bounds with 95% coverage [1]. *Abbreviations*: *Aβ42*, β-amyloid (1–42); *AD*, Alzheimer's disease; *QQ*, quantile-quantile; *pTau*, phosphorylated tau (181P); *tTau*, total tau

Reference

[1] Schützenmeister A, Jensen U, Piepho H-P. Checking normality and homoscedasticity in the general linear model using diagnostic plots. Commun Stat Simul Comput. 2012;41:141-54

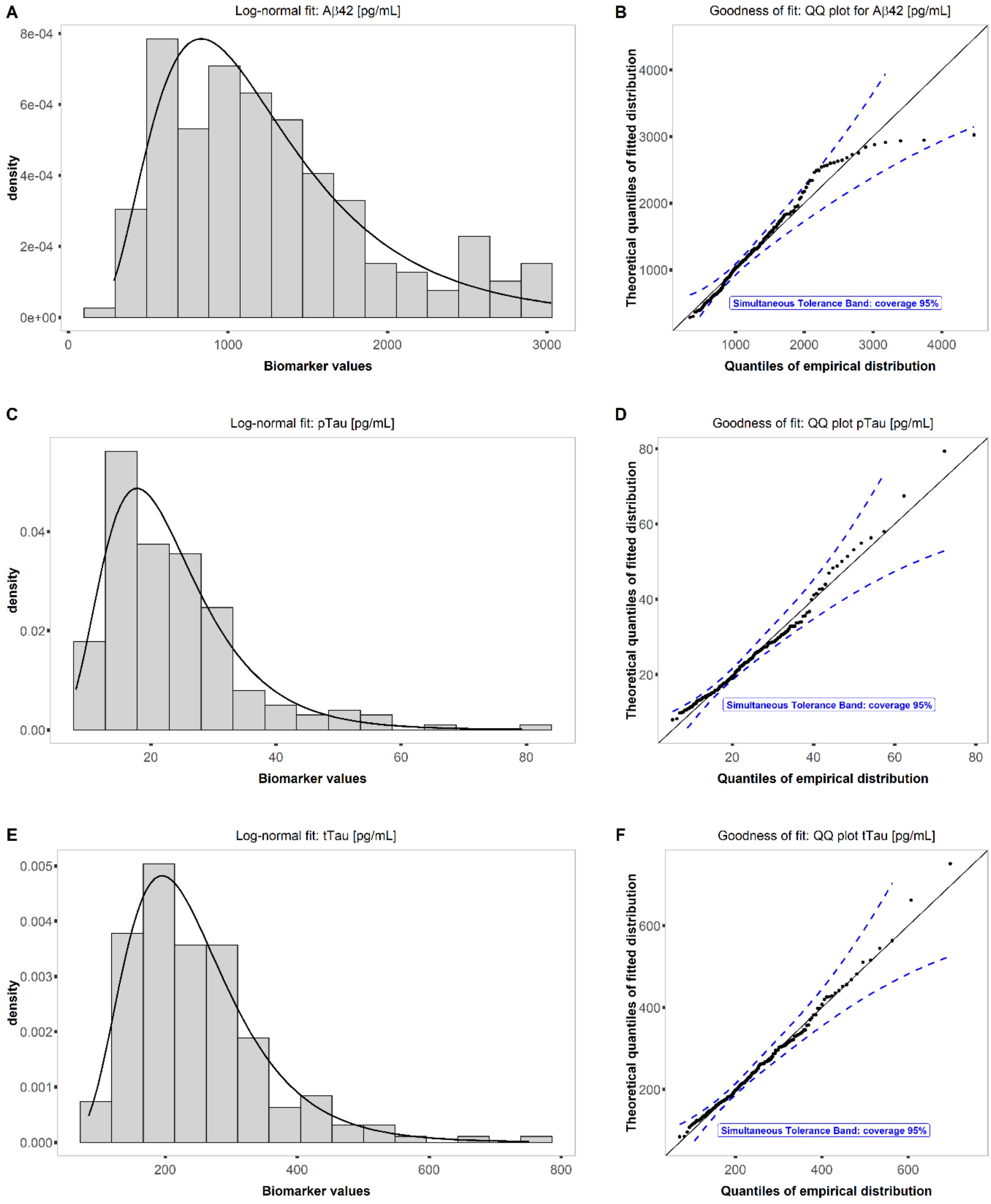

A
Log-normal fit: Aβ42 [pg/mL]
density
8e-04
6e-04
4e-04
2e-04
0e+00
0
1000
2000
3000
Biomarker values
B
Goodness of fit: QQ plot for Aβ42 [pg/mL]
Theoretical quantiles of fitted distribution
4000
3000
2000
1000
Simultaneous Tolerance Band: coverage 95%
1000
2000
3000
4000
Quantiles of empirical distribution
C
Log-normal fit: pTau [pg/mL]
density
0.04
0.02
0.00
20
40
60
80
Biomarker values
D
Goodness of fit: QQ plot pTau [pg/mL]
Theoretical quantiles of fitted distribution
80
60
40
20
Simultaneous Tolerance Band: coverage 95%
20
40
60
80
Quantiles of empirical distribution
E
Log-normal fit: tTau [pg/mL]
density
0.005
0.004
0.003
0.002
0.001
0.000
200
400
600
800
Biomarker values
F
Goodness of fit: QQ plot tTau [pg/mL]
Theoretical quantiles of fitted distribution
600
400
200
Simultaneous Tolerance Band: coverage 95%
200
400
600
Quantiles of empirical distribution
