# Supplementary material for: Elecsys CSF biomarker immunoassays demonstrate concordance with amyloid-PET imaging
Source: Alzheimers Res Ther. 2020 Mar 31;12:36. doi: 10.1186/s13195-020-00595-5 (PMC7110644; doi:10.1186/s13195-020-00595-5)
Supplement: Supplementary file 5 — CSF biomarkers versus PET SUVR for: (A) Aβ42 versus FLUTE SUVR, (B) Aβ42/Aβ40 versus FLUTE SUVR, (C) pTau/Aβ42 versus FLUTE SUVR, (D) Aβ42 versus FBP SUVR, (E) Aβ42/Aβ40 versus FBP SUVR and (F) pTau/Aβ42 versus FBP SUVR. [file 13195_2020_595_MOESM5_ESM.pdf]

Additional file 5: Supplementary Fig. S3 CSF biomarkers versus PET SUVR for: (A) Aβ42 versus FLUTE SUVR, (B) Aβ42/Aβ40 versus FLUTE SUVR, (C) pTau/Aβ42 versus FLUTE SUVR, (D) Aβ42 versus FBP SUVR, (E) Aβ42/Aβ40 versus FBP SUVR and (F) pTau/Aβ42 versus FBP SUVR. Lines in plots A, B, D and E represent the non-linear relationship between CSF biomarkers and SUVR. Lines in plots C and F represent the linear relationship between SUVR and pTau/Aβ42. Grey shaded areas represent the 95% CI from the model around the line. Grey dashed lines represent thresholds for SUVR (vertical) and CSF (horizontal) biomarkers. Red symbols represent Aβ-PET+; blue symbols represent Aβ-PET–; circles represent CN participants; triangles represent participants with MCI; squares represent participants with AD. Thresholds are: Aβ42, 1054 pg/mL; Aβ42/Aβ40, 64.0 (x0.001); pTau/Aβ42, 0.018. *Abbreviations*: *Aβ*, β-amyloid; *Aβ42*, β-amyloid (1–42); *Aβ42/Aβ40*, β-amyloid (1–42)/β-amyloid (1–40) ratio; *AD*, Alzheimer's disease; *CI*, confidence interval; *CN*, cognitively normal; *CSF*, cerebrospinal fluid; *FBP*, ^{18}F-Florbetapir; *FLUTE*, ^{18}F-Flutemetamol; *MCI*, mild cognitive impairment; *PET*, positron emission tomography; *pTau/Aβ42*, phosphorylated tau (181P)/β-amyloid (1–42) ratio; *SUVR*, standardised uptake value ratio

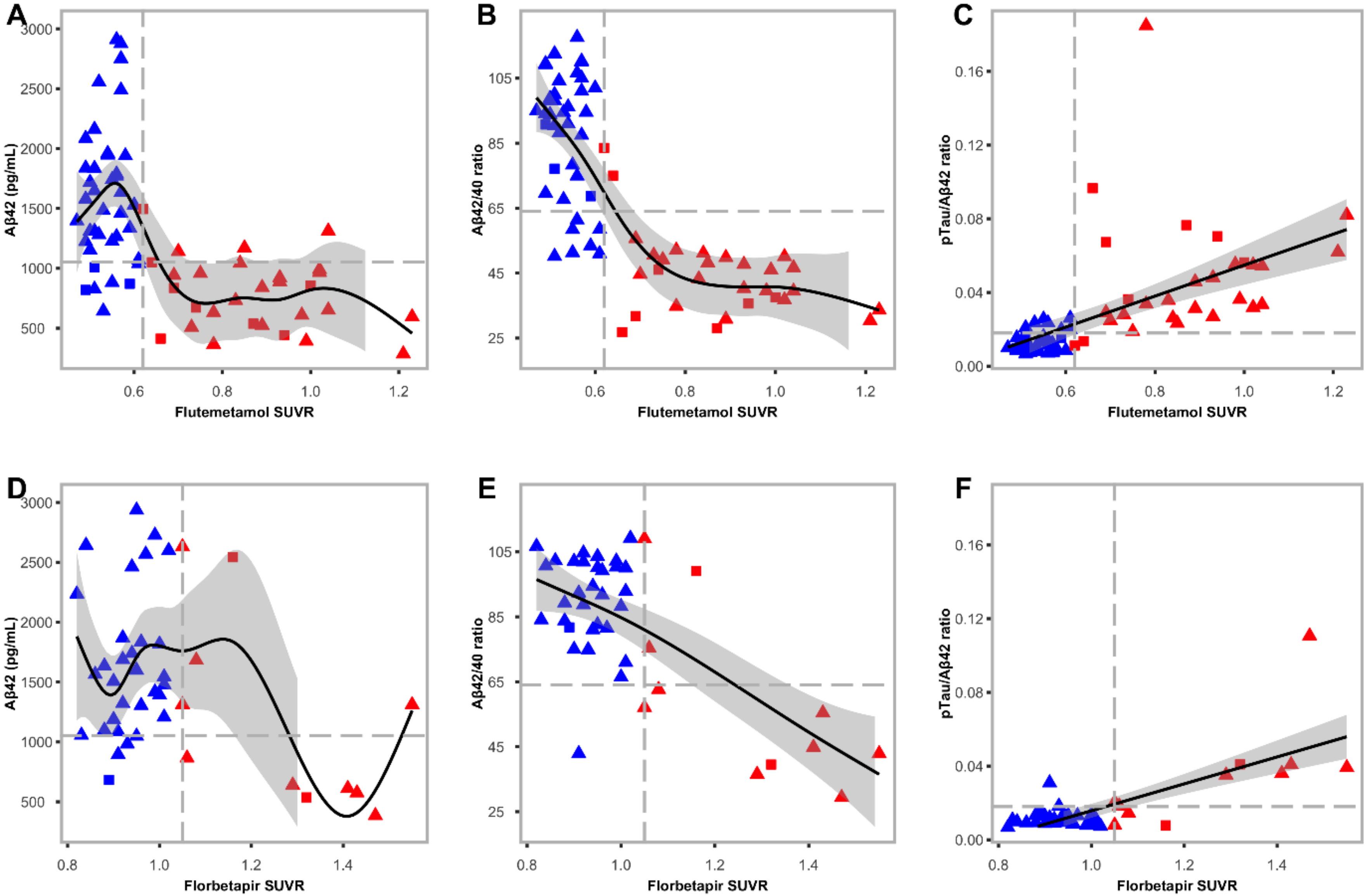
A
B
C
D
E
F
Aβ42 (pg/mL)
Aβ42/40 ratio
pTau/Aβ42 ratio
Flutemetamol SUVR
Florbetapir SUVR
