# Supplementary material for: Elecsys CSF biomarker immunoassays demonstrate concordance with amyloid-PET imaging
Source: Alzheimers Res Ther. 2020 Mar 31;12:36. doi: 10.1186/s13195-020-00595-5 (PMC7110644; doi:10.1186/s13195-020-00595-5)
Supplement: Supplementary file 6 — Results of ROC-AUC analysis – CSF biomarkers to predict Aβ-PET status, by PET tracer. [file 13195_2020_595_MOESM6_ESM.pdf]

Additional file 6: Supplementary Table S2 Results of ROC-AUC analysis – CSF biomarkers to predict Aβ-PET status, by PET tracer

Tracer	Biomarker	AUC (95% CI)	PPA (%)	NPA (%)	OPA (%)	Threshold
NAV/PiB	Aβ42	0.86 (0.79–0.94)	77	85	81	957 pg/mL
	tTau	0.82 (0.73–0.91)	86	72	79	215 pg/mL
	pTau	0.85 (0.77–0.93)	84	80	82	21.7 pg/mL
	Aβ42/Aβ40	0.94 (0.88–1.00)	93	91	92	0.0635
	tTau/Aβ42	0.95 (0.90–1.00)	91	98	94	0.234
	pTau/Aβ42	0.95 (0.90–1.00)	91	98	94	0.021
FLUTE	Aβ42	0.91 (0.84–0.97)	93	77	84	1198 pg/mL
	tTau	0.78 (0.66–0.89)	72	77	75	245 pg/mL
	pTau	0.81 (0.70–0.92)	76	79	78	23.5 pg/mL
	Aβ42/Aβ40	0.97 (0.94–1.00)	93	90	91	0.057
	tTau/Aβ42	0.96 (0.93–1.00)	86	95	91	0.258
	pTau/Aβ42	0.97 (0.93–1.00)	83	100	93	0.026
FBP	Aβ42	0.72 (0.51–0.94)	55	97	86	881 pg/mL

tTau	0.79 (0.64–0.95)	91	74	79	213 pg/mL
pTau	0.82 (0.07–0.95)	91	77	81	19.5 pg/mL
Aβ42/Aβ40	0.82 (0.63–1.00)	73	97	90	0.0646
tTau/Aβ42	0.84 (0.65–1.00)	82	94	90	0.184
pTau/Aβ42	0.84 (0.65–1.00)	82	94	90	0.015

Abbreviations: *Aβ*, β-amyloid; *Aβ42*, β-amyloid (1–42); *Aβ42/Aβ40*, β-amyloid (1–42)/β-amyloid (1–40) ratio; *AUC*, area under the curve; *CI*, confidence interval; *CSF*, cerebrospinal fluid; *FBP*, ^{18}F-Florbetapir; *FLUTE*, ^{18}F-Flutemetamol; *NAV*, ^{18}F-NAV4694; *NPA*, negative percentage agreement; *OPA*, overall percentage agreement; *PET*, positron emission tomography; *PiB*, ^{11}C-Pittsburgh compound B; *PPA*, positive percentage agreement; *pTau*, phosphorylated tau (181P); *pTau/Aβ42*, phosphorylated tau (181P)/β-amyloid (1–42) ratio; *ROC*, receiver operating characteristic; *tTau*, total tau; *tTau/Aβ42*, total tau/β-amyloid (1–42) ratio
