# Supplementary material for: Elecsys CSF biomarker immunoassays demonstrate concordance with amyloid-PET imaging
Source: Alzheimers Res Ther. 2020 Mar 31;12:36. doi: 10.1186/s13195-020-00595-5 (PMC7110644; doi:10.1186/s13195-020-00595-5)
Supplement: Supplementary file 7 — Results of ROC-AUC analysis – CSF biomarkers to predict Aβ-PET status in CN individuals. [file 13195_2020_595_MOESM7_ESM.pdf]

Additional file 7: Supplementary Table S3 Results of ROC-AUC analysis – CSF biomarkers to predict Aβ-PET status in CN individuals

Biomarker	AUC (95% CI)	PPA (%)	NPA (%)	OPA (%)	Threshold
Aβ42	0.84 (0.75–0.92)	71	87	83	1046 pg/mL
tTau	0.76 (0.66–0.85)	82	66	70	213 pg/mL
pTau	0.80 (0.71–0.88)	79	74	75	20.9 pg/mL
Aβ42/Aβ40	0.92 (0.86–0.98)	89	89	89	0.064
tTau/Aβ42	0.93 (0.87–0.99)	92	87	89	0.184
pTau/Aβ42	0.93 (0.87–0.99)	89	90	90	0.0186

Abbreviations: *Aβ*, β-amyloid; *Aβ42*, β-amyloid (1–42); *Aβ42/Aβ40*, β-amyloid (1–42)/β-amyloid (1–40) ratio; *AUC*, area under the curve; *CI*, confidence interval; *CN*, cognitively normal; *CSF*, cerebrospinal fluid; *NPA*, negative percentage agreement; *OPA*, overall percentage agreement; *PET*, positron emission tomography; *PPA*, positive percentage agreement; *pTau*, phosphorylated tau (181P); *pTau/Aβ42*, phosphorylated tau (181P)/β-amyloid (1–42) ratio; *ROC*, receiver operating characteristic; *tTau*, total tau; *tTau/Aβ42*, total tau/β-amyloid (1–42) ratio
